# Dual Activation of Phosphodiesterases 3 and 4 Regulates Basal Spontaneous Beating Rate of Cardiac Pacemaker Cells: Role of Compartmentalization?

**DOI:** 10.3389/fphys.2018.01301

**Published:** 2018-10-09

**Authors:** Tatiana M. Vinogradova, Evgeny Kobrinsky, Edward G. Lakatta

**Affiliations:** Laboratory of Cardiovascular Science, Intramural Research Program, NIA, NIH, Baltimore, MD, United States

**Keywords:** sinoatrial node cells, phosphodiesterases, PKA phosphorylation, L-type Ca^2+^ channel, sarcoplasmic reticulum, sarco(endo)plasmic reticulum calcium ATPase

## Abstract

Spontaneous firing of sinoatrial (SA) node cells (SANCs) is regulated by cyclic adenosine monophosphate (cAMP)-mediated, protein kinase A (PKA)-dependent (cAMP/PKA) local subsarcolemmal Ca^2+^ releases (LCRs) from ryanodine receptors (RyR). The LCRs occur during diastolic depolarization (DD) and activate an inward Na^+^/Ca^2+^ exchange current that accelerates the DD rate prompting the next action potential (AP). Basal phosphodiesterases (PDEs) activation degrades cAMP, reduces basal cAMP/PKA-dependent phosphorylation, and suppresses normal spontaneous firing of SANCs. The cAMP-degrading PDE1, PDE3, and PDE4 represent major PDE activities in rabbit SANC, and PDE inhibition by 3-isobutyl-1-methylxanthine (IBMX) increases spontaneous firing of SANC by ∼50%. Though inhibition of single PDE1–PDE4 only moderately increases spontaneous SANC firing, dual PDE3 + PDE4 inhibition produces a synergistic effect hastening the spontaneous SANC beating rate by ∼50%. Here, we describe the expression and distribution of different PDE subtypes within rabbit SANCs, several specific targets (L-type Ca^2+^ channels and phospholamban) regulated by basal concurrent PDE3 + PDE4 activation, and critical importance of RyR Ca^2+^ releases for PDE-dependent regulation of spontaneous SANC firing. Colocalization of PDE3 and PDE4 beneath sarcolemma or in striated patterns inside SANCs strongly suggests that PDE-dependent regulation of cAMP/PKA signaling might be executed at the local level; this idea, however, requires further verification.

## Introduction

**FIGURE 1:**
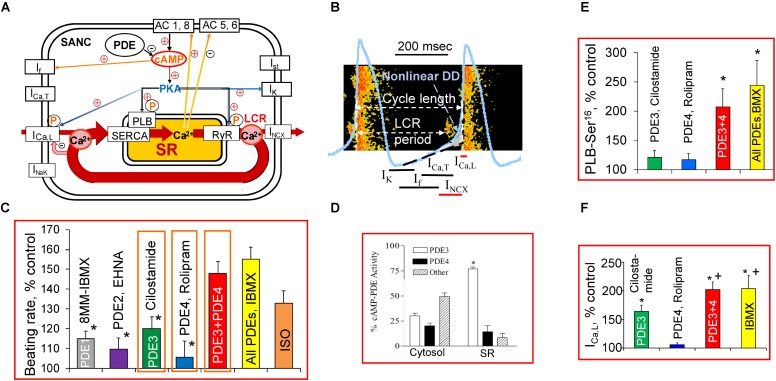
A schematic illustration of regulation of the basal cardiac pacemaker function by PDEs and PKA-dependent phosphorylation in intact SANC. **(A)** The coupled-clock pacemaker system. Intracellular SR Ca^2+^ cycling (in red) operates in conjunction with the ensemble of membrane ion channels on a beat-to-beat basis. Note that L-type Ca^2+^ channel and I_NCX_ current are both Ca^2+^ cycling proteins and surface membrane currents. Constitutive activation of the basal AC activity results in an elevated level of cAMP and cAMP/PKA-dependent phosphorylation, which is kept in check by a high basal PDE activity. The PKA-dependent phosphorylation modulates function of Ca^2+^ cycling proteins (PLB, RyR, L-type Ca^2+^ channel, NCX current). **(B)** Schematic illustration of spontaneous SANC APs, Ca^2+^ transients, LCRs, and several major ion currents involved in generation of the DD. The LCR-induced increase in local [Ca^2+^] beneath the sarcolemma activates an inward NCX current creating exponential increase in the DD rate (Non-linear DD). See text for additional details. **(C)** Comparison of relative changes in the spontaneous SANC beating rate produced by selective inhibitors of PDE1-PDE4 alone or in combination and expressed as % of control. One-way ANOVA with Tukey *post hoc* test, ^∗^*P* < 0.05 vs. PDE3 + PDE4 or All PDEs inhibition. **(D)** Distribution of PDE3 and PDE4 activities in SR-enriched and cytosolic fractions in rabbit SA node [modified from Shakur et al. (2002)]. **(E)** Average changes in PLB phosphorylation at Ser^16^ site in rabbit SANC by cilostamide (0.3 μmol/L) or rolipram (2 μmol/L) alone, combination of cilostamide + rolipram or 100 μmol/L IBMX expressed as % of control (*n* = 7–9 rabbits). One-way ANOVA with Tukey *post hoc* test, ^∗^*P* < 0.05 vs. cilostamide or rolipram alone. **(F)** Regulation of L-type Ca^2+^ current amplitude by dual PDE3 + PDE4 activation; average increases in I_Ca,L_ amplitude during inhibition of PDE3 or PDE4 alone, concurrent PDE3 + PDE4 inhibition, or IBMX presented as % of control (*n* = 5–7 SANC). One-way ANOVA with Bonferroni *post hoc* test ^∗^*P* < 0.01 vs. rolipram alone; ^+^*P* < 0.01 vs. cilostamide alone. **(C,E,F)** Modified from Vinogradova et al. (2008, 2018).

The sinoatrial (SA) node, the primary physiological pacemaker of the heart, drives more than 3 billion heartbeats during a human life span. The SA node automaticity is generated within the SA node pacemaker cells (SANCs), which fire spontaneous action potentials (APs) because of gradual depolarization of the membrane potential called diastolic depolarization (DD) linked to complex interactions of ‘coupled clock’ mechanisms. The ‘membrane clock’ refers to multiple voltage-gated ion channels and transporters in the cell membrane, including hyperpolarization-activated “funny” current I_f_, L-type and T-type Ca^2+^ currents (I_Ca,L_ and I_Ca,T_), delayed rectifier potassium current (I_K_), Na^+^/Ca^2+^ exchange current (I_NCX_), Na^+^/K^+^ exchange current (I_NaK_), etc. (**Figures 1A,B**; Irisawa et al., 1993; Mangoni and Nargeot, 2008).

Like other cardiac cells, SANCs have the sarcoplasmic reticulum (SR) and are equipped to cycle Ca^2+^ via sarco/endoplasmic reticulum Ca^2+^-ATPase (SERCA2) and Ca^2+^ release channels, ryanodine receptors (RyRs). The SANCs can generate spontaneous local Ca^2+^ releases (LCRs) from RyR in the subsarcolemmal space during late DD before the AP upstroke (**Figures 1A,B**; Bogdanov et al., 2001). Numerous studies have confirmed the presence of rhythmic LCRs under normal physiological conditions in SANCs of different species (Huser et al., 2000; Lipsius et al., 2001; Vinogradova et al., 2004; Joung et al., 2009; Wu et al., 2009; Sirenko et al., 2017). The LCRs activate an inward I_NCX_, which exponentially accelerates the rate of DD, prompting the “Membrane clock” to generate the next AP (Bogdanov et al., 2001; Sanders et al., 2006; Lakatta et al., 2010). Colocalization of Na^+^/Ca^2+^ exchanger (NCX) and RyRs in rabbit SANC (Lyashkov et al., 2007) permits a quick conversion of LCRs beneath the sarcolemma into changes in the inward current that depolarizes the membrane potential. The SR-generated LCRs can occur independent of concurrent changes in the membrane potential; they persist during the voltage clamp of the cell membrane or in permeabilized SANCs (Vinogradova et al., 2004; Lakatta et al., 2010), manifesting the intracellular SR Ca^2+^ cycling “Ca^2+^ clock” in the absence of the “Membrane clock.” The dynamic interaction of the “Ca^2+^ clock” and “Membrane clock” permits a high level of mutual entrainment between two individual clocks on a beat-to-beat basis (**Figures 1A,B**). This clock entrainment provides an additional degree of flexibility and robustness to the generation of spontaneous APs by the cardiac pacemaker cells (Lakatta et al., 2010; Yaniv et al., 2015).

The cyclic adenosine monophosphate (cAMP) is a ubiquitous secondary messenger that modulates multiple cell processes, e.g., cAMP-mediated protein kinase A (PKA)-dependent protein phosphorylation. Basal level of cAMP in rabbit SANCs is substantially higher than in ventricular myocytes (VM) (Vinogradova et al., 2006; Younes et al., 2008; Lakatta et al., 2010) due to constitutive activation of adenylyl cyclases (ACs). This basal AC activity is independent of constitutive β-adrenergic receptor (β-AR) activation, since neither the β1-AR antagonist, CGP-20712A, nor the β2-AR inverse agonist, ICI 118,551 affect the spontaneous SANCs beating rate (Vinogradova et al., 2006; Lakatta et al., 2010). Both the “Membrane clock” and “Ca^2+^ clock” are regulated by cAMP and cAMP-mediated PKA-dependent phosphorylation. Funny current is directly activated by cAMP (DiFrancesco and Tortora, 1991; St Clair et al., 2013). Several ion currents in SANCs are targets of the PKA-dependent phosphorylation including I_Ca,L_, I_K_, I_f_, etc. (Irisawa et al., 1993; Mangoni and Nargeot, 2008; Liao et al., 2010). Proteins involved in the intracellular SR Ca^2+^ cycling in SANC [i.e., phospholamban (PLB), RyR, and SERCA] are also regulated by PKA-dependent phosphorylation (Vinogradova et al., 2006; Lakatta et al., 2010). The generation of rhythmic spontaneous LCRs and basal spontaneous firing of SANCs require a high basal level of cAMP and cAMP-mediated PKA-dependent phosphorylation (Vinogradova et al., 2006; Lakatta et al., 2010).

The cell cAMP level is the result of a balance between cAMP production by ACs and its degradation into 5′-AMP by cyclic nucleotide phosphodiesterases (PDEs), the only known mechanism to degrade cAMP (Beavo and Brunton, 2002). The PDE superfamily contains 11 distinct gene families (PDEs 1–11), and at least four PDE families (PDE1-PDE4) can hydrolyze cAMP in the heart. Specifically, PDE1 is activated by Ca^2+^/calmodulin, PDE2 is stimulated by cGMP, PDE3 is inhibited by cGMP, and PDE4 is specific for cAMP. The PDE3 and PDE4 represent the major cAMP PDE activities in cardiac myocytes (Takahashi et al., 2002; Wechsler et al., 2002; Mongillo et al., 2004).

The normal AP firing of rabbit SANC is regulated by basal PDE activation, and its inhibition by broad-spectrum PDE inhibitor, 3-isobutyl-1-methylxanthine (IBMX), markedly increases the level of cAMP and concurrently increases the spontaneous SANC firing rate by ∼50%, which surpasses the positive chronotropic effect of the β-AR agonist isoproterenol (Vinogradova et al., 2008; **Figure 1C**). Combined activities of PDE3 and PDE4 represent the major basal PDE activities in the rabbit SA node, accounting for ∼50% in cytosolic and ∼90% in SR fractions (**Figure 1D**; Shakur et al., 2002). A synergistic relationship between PDE3 and PDE4 inhibition has been noted in different cell types, including vascular smooth muscle cells (Palmer et al., 1998), brown adipose tissue (Kraynik et al., 2013), and rat VM (Mika et al., 2013). Normal automaticity of rabbit SANC is regulated by dual PDE3 + PDE4 activation apparently operating in a synergistic manner (Vinogradova et al., 2018).

This mini-review is focused upon how cAMP-degrading PDEs regulate the normal spontaneous beating rate of SANCs, including expression and distribution of different PDE subtypes within SANCs, specific targets, and mechanisms of PDE-dependent regulation of spontaneous SANC firing. Evidence for compartmentalization of cAMP signaling in cardiac pacemaker cells under basal conditions is also discussed.

## Basal PDE Activity Controls Normal Spontaneous Firing of Cardiac Pacemaker Cells

Suppression of PDE activity in isolated SA node produces an increase in cAMP level (Shahid and Rodger, 1989), acceleration of DD rate, and increase in the spontaneous SA node beating rate of different species (Kodama et al., 1983; Orito et al., 1996; Liu et al., 2011; Sharpe et al., 2017). The PDE3 is the most abundant PDE isoenzyme in the myocardial tissue of most mammalian species (Osadchii, 2007). Although PDE3 can hydrolyze both cAMP and cGMP, the catalytic rates for cAMP are 5–10-fold higher, than for cGMP, which makes PDE3 very efficient in degrading cAMP (Bender and Beavo, 2006; Osadchii, 2007).

The PDE3 inhibition increases the spontaneous beating rate of the SA node in guinea pigs (Orito et al., 1996), rabbits (Kaumann et al., 2009), dogs (Sato et al., 1986), and humans (Jaski et al., 1985). In the murine heart, PDE4 is the major PDE isoform and accounts for∼60% of the total cAMP hydrolyzing activity (Osadchii, 2007). Inhibition of either PDE3 or PDE4 increases the spontaneous beating of the mouse SANCs (Hua et al., 2012) or rat SA node (Kaumann et al., 2009).

The PDE1 is an abundant cytosolic PDE isoenzyme in human ventricular myocardium (Wallis et al., 1999) or VM (Johnson et al., 2012). Targets of PDE1-dependent regulation in VM, however, remain obscure, since inhibition of PDE1 activity produces a decrease rather than increase in contraction amplitude of human VM (Johnson et al., 2012). Nimodipine-sensitive activity of PDE1, measured in lysates of isolated rabbit SANCs, accounted for ∼40% of total PDE activity (Lukyanenko et al., 2016), but PDE1 inhibition increased spontaneous firing of rabbit SANCs by ∼15% (**Figure 1C**). The PDE1 activity might have a greater impact at higher cAMP levels; indeed, stimulation of ACs with forskolin markedly increases both cAMP level and PDE1 activity in paced mouse VM (Sprenger et al., 2016).

Although average increases in the basal spontaneous beating rate of rabbit SANCs by inhibition of single cAMP-degrading PDEs (PDE1–PDE4) are relatively small (**Figure 1C**), concurrent inhibition of PDE3 + PDE4 increases the spontaneous SANC beating rate by ∼48% (Vinogradova et al., 2018), creating an effect comparable with that of IBMX (**Figure 1C**). An acceleration of spontaneous SANC firing by concomitant PDE3 + PDE4 inhibition by ∼twofold exceeds the summed increases in the spontaneous firing produced by inhibition of PDE3 (∼20%) and PDE4 (∼5%) alone (Vinogradova et al., 2018), indicating that the dual PDE3 + PDE4 activation operates synergistically to suppress basal spontaneous firing of rabbit SANCs (Vinogradova et al., 2018).

## Effects of PDE Inhibition on Protein Phosphorylation in SANC

An increase in cAMP-mediated PKA-dependent phosphorylation of multiple proteins in cardiac cells occurs in response to PDE inhibition. Among Ca^2+^ cycling proteins phosphorylated in the basal state in rabbit SANC are PLB (Vinogradova et al., 2006; Lakatta et al., 2010), RyRs (Li et al., 2016), and likely L-type Ca^2+^ channels (Petit-Jacques et al., 1993). PLB modulates kinetics of SR Ca^2+^ pumping: in unphosphorylated state PLB colocalizes with SERCA2 to inhibit its function to pump Ca^2+^ into SR (MacLennan and Kranias, 2003). Phosphorylation of PLB by PKA at Ser^16^ site in VM relieves this inhibition elevating SERCA activity by ∼2–3-fold (MacLennan and Kranias, 2003). Phosphorylation status of PLB at Ser^16^ site is a useful marker of PKA-dependent protein phosphorylation in SANC. Inhibition of either PDE3 or PDE4 alone produces only minor (∼20%, *P* > 0.05) increase in PLB phosphorylation at Ser^16^ site in SANC, but dual PDE3 + PDE4 inhibition increases PLB phosphorylation by ∼110%, an effect comparable to that of IBMX (**Figure 1E**). Therefore, basal PLB phosphorylation at Ser^16^ site in SANC appeared to be regulated by synergism of concurrent PDE3 + PDE4 activation (Vinogradova et al., 2018). This boost in basal PKA-dependent phosphorylation, produced by dual PDE3 + PDE4 inhibition and reflected in PLB phosphorylation, might also affect multiple other proteins involved in the regulation of cardiac pacemaker function which require further investigation.

## Effects of PDE Inhibition on ionic Currents and SR Ca^2+^ Cycling in SANC

The L-type Ca^2+^ channels are a well-known target of cAMP-mediated PKA-dependent pathway regulated by PDE activation. Comparable increases in basal I_Ca,L_ amplitude by ∼60 and ∼72% occur when PDE3 or PDE4 are inhibited in mouse SANC (Hua et al., 2012), consistent with an important role of basal PDE3 and PDE4 activity in the murine heart (Osadchii, 2007). Synergistic effect of dual PDE3 + PDE4 inhibition on I_Ca,L_ amplitude was observed both in human and rabbit atrial myocytes, creating effect comparable to that of IBMX. In contrast, PDE4 inhibition alone is without effect and PDE3 inhibition only moderately increases I_Ca,L_ amplitude in human and rabbit atrial myocytes (Kajimoto et al., 1997). The PDE4 inhibition in rabbit SANC, as in human atrial myocytes, has no effect on I_Ca,L_ amplitude, while inhibition of PDE3 increases I_Ca,L_ by ∼60% (**Figure 1F**). Dual PDE3 + PDE4 inhibition increases I_Ca,L_ in rabbit SANC by ∼100%, markedly exceeding combined effects of separate PDE3 or PDE4 inhibition and creating effect comparable with that of IBMX (**Figure 1F**). Therefore, dual PDE3 + PDE4 activation regulates basal I_Ca,L_ amplitude in rabbit SANC in a synergistic manner (Vinogradova et al., 2018).

Other ionic currents involved in the generation of DD are also regulated by PDEs, e.g., inhibition of PDE3 in rabbit SANC increases I_K_ and shifts voltage dependence of I_f_ activation to more positive potentials (DiFrancesco and Tortora, 1991; Hata et al., 1998; Vinogradova et al., 2008). In mouse SANC, inhibition of PDE activity by IBMX or PDE4 activity by rolopram shifts voltage dependence of I_f_ current to more positive potentials (St Clair et al., 2017). The PDE3 inhibitor, milrinone, significantly increases I_f_ current amplitude by ∼20% (Springer et al., 2012) without shift of the voltage dependence of I_f_ current (St Clair et al., 2017).

The LCRs are also regulated by basal PDE activation both in intact and permeabilized rabbit SANCs (Vinogradova et al., 2008, 2018; Lakatta et al., 2010). During each spontaneous cycle, AP-induced Ca^2+^ influx through L-type Ca^2+^ channels triggers global Ca^2+^ transient, depleting SR Ca^2+^, resetting the “Ca^2+^ clock,” and leading to LCR termination. When the SR Ca^2+^ content is refilled by SERCA, LCRs start to occur, and the time from AP-induced Ca^2+^ transient to the onset of LCRs is the LCR period (**Figure 1B**). An increase in cAMP-mediated PKA-dependent phosphorylation of Ca^2+^ cycling proteins concurrently elevates amount of Ca^2+^ (influx via I_CaL_) available for pumping into SR, accelerates the SR Ca^2+^ refilling (PLB), and likely alters the threshold for spontaneous Ca^2+^ releases (RyR), creating conditions required to boost spontaneous LCRs and speed up their appearance.

In intact rabbit, SANCs inhibition of PDE3 markedly increases the LCR size and number per each spontaneous cycle by ∼20% each (*P* < 0.05) and decreases the LCR period by ∼15% (*P* < 0.05), while changes in these parameters by rolipram are relatively small. Dual PDE3 + PDE4 inhibition, however, produces a synergistic effect and augments both the LCR size and number by ∼45% (*P* < 0.01) each, as RyR activation becomes more synchronized via RyR recruitment and decreases the LCR period by ∼40% (*P* < 0.01). An amplification of local RyR Ca^2+^ release activates augmented I_NCX_ at earlier times leading to an increase in the DD rate and spontaneous SANC beating rate (Vinogradova et al., 2018).

The contribution of “funny” current in the acceleration of spontaneous SANC beating rate by dual PDE3 + PDE4 inhibition was assessed in the presence or absence of I_f_ current inhibitors. Suppression of I_f_ current by either ivabradine or Cs^+^ markedly decreased the spontaneous SANC beating rate. The positive chronotropic effect of dual PDE3 + PDE4 inhibition or IBMX, however, remained preserved even in the absence of I_f_ current (Vinogradova et al., 2018), indicating that the I_f_ current was not indispensable for the positive chronotropic effect of dual PDE3 + PDE4 inhibition. This might be related to specific locations of I_f_ channels within lipid raft domains of rabbit SANC (Barbuti et al., 2004), which could provide spatial barriers shielding I_f_ channels from cAMP elevation produced by dual PDE3 + PDE4 inhibition.

In contrast, when RyR function is disabled by ryanodine, dual PDE3 + PDE4 inhibition failed to accelerate the DD rate or increase the spontaneous SANC beating rate (Vinogradova et al., 2018), despite preserved increase of I_Ca,L_ and I_K_ amplitudes (Vinogradova et al., 2008), indicating requirement of intact RyR function. Therefore, effects of PDE inhibition to increase function of ionic currents alone are not sufficient to increase the basal spontaneous SANC beating rate, which requires a link between PDE inhibition-induced increases in ionic currents and SR Ca^2+^ cycling within the coupled clock system (**Figures 1A,B**).

**FIGURE 2:**
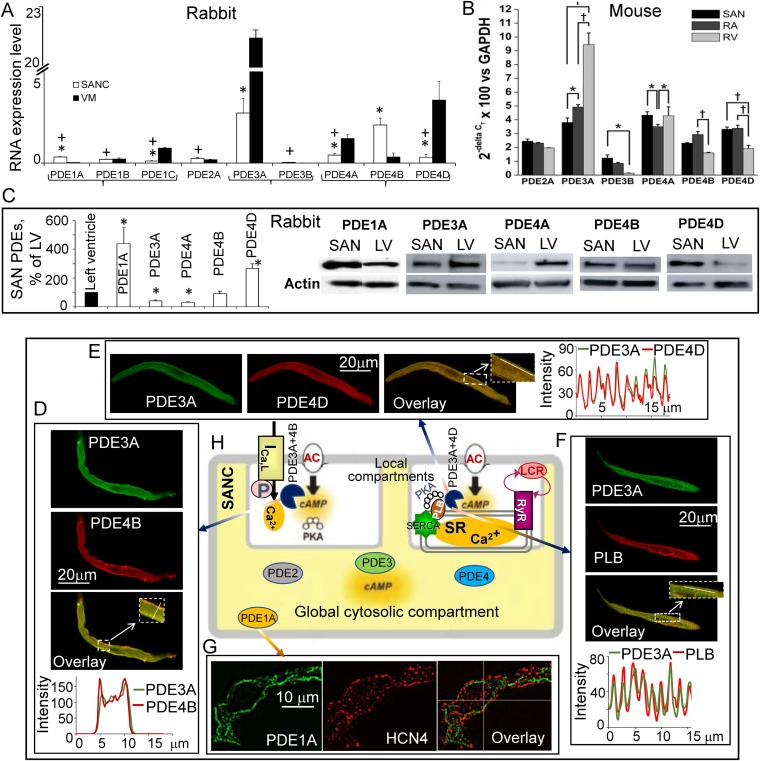
Expression of cAMP-degrading PDEs at RNA and protein levels in the cardiac pacemaker and ventricle. Possible organization of PDE1A and major PDE3 and PDE4 subtypes within compartments in single rabbit SANC. **(A)** Relative expression of PDE-coding transcripts (mean ± SEM) in rabbit SANCs and VM; (*n* = 4–9); 1-way ANOVA with Tukey *post hoc* test, adjusted ^∗^*P* < 0.05 (SANC vs. VM for each PDE subtype); ^+^*P* < 0.05 (PDE subtypes in SANC vs. PDE3A or PDE4B); **(B)** Quantitative mRNA expression of PDE 2, 3, and 4 subtypes in mouse SA node, right atrium (RA) and right ventricle (RV). Expression of PDE2A, PDE3A, PDE3B, PDE4A, PDE4B, and PDE4D are shown relative to GAPDH as (mean ± SEM); *n* = 5 SA node trials, 5 RA trials, and 3 RV trials; ^∗^*P* < 0.05; ^+^*P* < 0.001 by two way ANOVA with Tukey’s *post hoc* test [modified from Hua et al. (2012)]. **(C)** (left) average data (*n* = 8) of PDE1A, PDE3A, PDE4A, PDE4B, and PDE4D protein expression in the rabbit SA node compared with the left ventricle (LV = 100%), column statistics ^∗^*P* < 0.05; (right) representative Western blots of major PDE subtypes in the rabbit SA node and left ventricle. **(D)** Double immunostaining for PDE3A, PDE4B, and superimposed images (inset shows magnification of the rectangular area in overlay); intensity plots (taken along the line in inset) show overlapping distribution of PDE3A with PDE4B beneath sarcolemma of SANC. **(E)** Double immunostaining for PDE3A, PDE4D, and merged images (inset shows magnification of the rectangular area in overlay); intensity plots (taken along the line in inset) display overlapping distribution of PDE3A with PDE4D along Z-lines in SANC. **(F)** Double immunostaining for PDE3A, PLB, and merged images (inset shows magnification of the rectangular area in overlay); intensity plots (taken along the line in inset) display overlapping distribution of PDE3A and PLB. **(G)** Double immunostaining for PDE1A, HCN4, and merged images. **(H)** A schematic of possible organization of compartmentalized signaling in rabbit SANC associated with L-type Ca^2+^ channels or PLB-dependent regulation of SR Ca^2+^ cycling. **(A,C–G)** modified from Vinogradova et al. (2018) and Lukyanenko et al. (2016).

Though L-type Ca^2+^ channels or PLB and likely others are regulated by concurrent PDE3 + PDE4 activation in a synergistic manner (**Figure 1**), changes in global intracellular cAMP in SANC did not follow this pattern. Specifically, dual PDE3 + PDE4 inhibition in SANC lysates increases cAMP level by only ∼90% less than the effect produced by IBMX (∼160%) or the sum of cAMP elevations created by inhibition of either PDE3 (∼45%) or PDE4 (∼56%) alone (Lukyanenko, unpublished data). Therefore, an increase in the spontaneous SANC beating rate by dual PDE3 + PDE4 inhibition (**Figure 1C**) is not created by changes in global cAMP, but likely by changes in local cAMP levels produced in the vicinity of PLB or L-type Ca^2+^ channels (scheme in **Figure 2**) or RyR etc.

Rabbit SANC lack t-tubular system, but they have considerable number of caveolae, flask-like invaginated lipid rafts containing caveolin, which could provide abundant physical boundaries for localized cAMP signaling. In rabbit SANCs, caveolae increase the surface plasma membrane by ∼115 and ∼30% in VM (Masson-Pevet et al., 1980). A variety of signaling molecules could be targeted to caveolae, including GPCRs, ACs, and PKA (Rybin et al., 2000; Younes et al., 2008; Bhogal et al., 2018). The subpopulation of L-type Ca^2+^ channels and HCN4 channels are also localized to caveolae (Barbuti et al., 2004; Glukhov et al., 2015). Caveolae have been identified as membrane subdomains that compartmentalize β-adrenergic receptor signaling and as negative regulators of cAMP accumulation in cardiac myocytes (Rybin et al., 2000; Bhogal et al., 2018). Though activity of PDE3 and PDE4 in rabbit SA node has been measured (**Figure 1D**), there is no information on how much of that activity might be inside or outside of caveolae.

## Evidence for Compartmentalized cAMP-PKA Signaling in VM

In VM, intracellular cAMP concentration in the basal conditions is close to 1 μmol/L, and this value is ∼10-fold higher during hormone or neurotransmitter activation (Iancu et al., 2008; Borner et al., 2011; Koschinski and Zaccolo, 2017). Multiple PDEs are expressed in each cell, with different affinities for cAMP: PDE3 in the range of 10–100 nmol/L (Manganiello et al., 1995), PDE4 in the range of 2–8 μmol/L (Salanova et al., 1998), while affinity of PDE1A or PDE2 exceeds 10 μmol/L (Bender and Beavo, 2006). Thus, cAMP is degraded over a wide range of concentrations and cells can maintain cAMP level at the physiological range both in the basal state or during hormone and neurotransmitter stimulation.

Because affinity of endogenous PKA for cAMP is in the range 100–300 nmol/L (Mongillo et al., 2004) and free diffusion of cAMP within the cell is relatively fast (∼200 μm^2^/s) (Saucerman et al., 2014), cAMP would rapidly spread, PKA would be fully activated under basal conditions, and hormones would not be able to produce any cAMP-mediated PKA-dependent functional responses. This controversy led to a hypothesis that PKA is compartmentalized in special domains with significantly lower basal cAMP level compared with that of the global cytosol (Iancu et al., 2007). Therefore, intracellular pools of cAMP in the cell, their signaling pathways, and functional responses are spatially and functionally compartmentalized by PDEs, which rapidly degrade cAMP, providing functional barriers to cAMP diffusion (Bender and Beavo, 2006; Houslay, 2010; Francis et al., 2011; Keravis and Lugnier, 2012; Conti et al., 2014). The PDEs might create local pools “microdomains” with high or low cAMP levels; in the latter case, PDEs act like “black holes” converting cAMP into 5′-AMP and thus, protecting specific compartments from cAMP influx and PKA activation (Conti et al., 2014; Maurice et al., 2014).

Genetic manipulations of mice showed that both PDE3 and PDE4 may reside in the same localized compartments associated with either L-type Ca^2+^ channels or SR in VM. Indeed, PDE3A is colocalized with SERCA2-PLB-AKAP18 multiprotein complex or “signalosome” that regulates refilling of SR through modulation of PLB phosphorylation both in mouse and human VM (Ahmad et al., 2015). In the mouse heart, PDE4B is a part of the L-type Ca^2+^ channel complex and represents the major PDE isoform modulating I_Ca,L_ amplitude during β-AR stimulation (Leroy et al., 2011). The PDE4D is incorporated in the SERCA2-PLB signaling complex in mouse VM (Kerfant et al., 2007), while PDE4D3 was integrated into SR-associated RyR2 complex (Lehnart et al., 2005).

## Rna Abundance, Protein Expression, and Distribution of Different Pde Subtypes in Sanc

At the messenger RNA level, PDE3A, PDE4A, PDE4B, and PDE4D are the major cAMP-degrading PDE subtypes expressed in both rabbit SANC and VM (Vinogradova et al., 2018). Expressions of PDE3A and PDE4B mRNA in rabbit SANC are comparable and exceed expression of other PDE subtypes (**Figure 2A**). Compared with PDE3 or PDE4, PDE1 mRNA in rabbit SANC is relatively low, but PDE1A transcript abundance in SANC surpasses that in VM by fourfold (Lukyanenko et al., 2016). Interestingly, mRNA transcripts for PDE2A, PDE3A, PDE4A, PDE4B, and PDE4D are comparably expressed in the mouse SA node (**Figure 2B**; Hua et al., 2012). Expression of PDE3A and PDE4A protein was less abundant in the rabbit SA node compared with the left ventricle; expression of PDE4B protein was similar in both tissues, while expression of PDE4D (Vinogradova et al., 2018) and PDE1A protein (Lukyanenko et al., 2016) was significantly higher in the rabbit SA node than in ventricle (**Figure 2C**).

Very little is known about the distribution of major PDE subtypes within SANC. Recent studies established that PDE1A, PDE3A, and PDE4B are localized beneath the sarcolemma of rabbit SANC (**Figure 2**; Lukyanenko et al., 2016; Vinogradova et al., 2018). Colocalization of PDE3A and PDE4B beneath the sarcolemma of rabbit SANC (**Figure 2D**) suggests that, like in the mouse heart, these PDE subtypes could work together limiting Ca^2+^ influx through L-type Ca^2+^ channels in a synergistic manner (**Figure 1F**). The PDE3A is also detected in a striated pattern and colocalizes with the *Z*-line associated protein α-actinin in rabbit SANC. Similar to human or mouse VM (Ahmad et al., 2015), PDE3A is colocalized with SERCA, PLB, and PDE4D in striated patterns inside SANCs (Vinogradova et al., 2018; **Figure 2**). Colocalization of PDE3A and PDE4D with SERCA and PLB suggests that these PDE isoforms could likely regulate cAMP-mediated PKA-dependent phosphorylation of major SR proteins in SANC (**Figure 2**).

## Synergistic Effects of Dual Pde3 + Pde4 Inhibition in Sanc Are Likely Executed at the Local Level

Though dual PDE3 and PDE4 inhibition could interact synergistically to modulate functional effects mediated by cAMP in multiple cell types, the mechanisms underlying these synergistic effects remain unclear. This synergistic effect could be based on colocalization and interaction of different PDE3 and PDE4 subtypes (**Figure 2**). Since PDE3 affinity is ∼10–100 nmol/L, it is likely that PDE3 is active and degrades cAMP in the basal state, while PDE4 remains dormant. An increase in local cAMP level by inhibition of PDE3 alone may increase cAMP to a range required for PDE4 activation, concurrently elevating PKA-dependent phosphorylation of PDE4, which is associated with 2–6-fold increase in PDE4 activity (Sette and Conti, 1996). Activation of PDE4 would lead to a more efficient degradation of cAMP, creating a PKA-mediated feedback loop to promote local cAMP degradation. Therefore, PDE3- and PDE4-dependent modulation of spontaneous beating of SANC can be self-adaptive, i.e., the full functional effect being achieved only when both PDE3 and PDE4 are concurrently inhibited, elevating local level of cAMP and PKA-dependent phosphorylation to their highest levels and leading to a full-sized synergistic functional response. Coordinated regulation of several targets (L-type Ca^2+^ channels, PLB, etc.) by synergistic dual PDE3 + PDE4 activation could be energetically beneficial, since slight variations in local cAMP levels at multiple locations could lead to substantial functional effects.

Recent studies demonstrated that physiologically relevant cAMP signals operate within the nanometer range, creating local cAMP nanodomains, while ‘global cAMP’ is less involved in functional responses (Surdo et al., 2017). Computational models of cAMP signaling help to understand changes in cAMP activity at the local level in the subcellular compartments of cardiac cells. Models predict that low basal [cAMP] in caveolae is critically dependent on restricted cAMP diffusion between membrane compartments and cytosol (Iancu et al., 2007). The cAMP gradients might be also shaped by enhanced PDE activity (Saucerman et al., 2014), as e.g., in rabbit SANC. Future numerical models might explain how specific patterns of different PDE3 and PDE4 subtype protein expressions in rabbit SANC could impact local and global cAMP levels and affect numerous players involved in generation of the cardiac pacemaker function.

## Future Directions in Measurements of Local cAMP-PKA Signaling in SANC

Though it is helpful to directly measure PDE activity using classical biochemical assays, this experimental approach lacks spatial resolution. Local degradation of cAMP produced by PDEs creates cAMP gradients and forms nanodomain organization of cellular signaling (Lohse et al., 2017). Real-time imaging of changes in [cAMP] dynamics using fluorescence resonance energy transfer (FRET)-based reporters is a powerful tool to study local intracellular signaling events linked to PDE activity. Multiple FRET sensors have been constructed to image local changes in cAMP in living cells based mostly on interaction between pairs of green (GFP) and yellow (YFP) fluorescent proteins. Upon cAMP binding, the conformation of FRET sensor protein changes, leading to displacement of fluorophores and alteration of FRET signal. Though FRET imaging has enhanced our understanding of compartmentalized cAMP signaling in different cell types, i.e., cardiomyocytes, pancreatic-β cells, neuron, and cancer cells (Henderson et al., 2014; Larsen et al., 2016; Maiellaro et al., 2016; Beltejar et al., 2017; Elliott et al., 2017), employment of FRET in the cardiac pacemaking field has only recently begun to emerge (Yaniv et al., 2015). Exploration of local signaling in cardiac pacemaker cells using FRET sensors, however, is a challenging task, because expression of detectable level of FRET sensors requires a culture of SANC. Even a short-term culture markedly changes basal characteristics of rabbit SANC via significant decrease in the level of type 2 regulator of G-protein signaling (RGS2) that facilitates activation of the AC/cAMP/PKA pathway via G_i_ inhibition, leading to a diminished level of cAMP/PKA-dependent phosphorylation accompanied by a ∼50% decrease in spontaneous SANC firing rate (Yang et al., 2012). Furthermore, cultured SANCs lose their spindle shape and became spherical or spread out with more than three projections (Yang et al., 2012). Changes in the cell shape alter the surface-to-volume ratio and modify the local balance of cAMP synthesis and degradation (Saucerman et al., 2014). Because of substantial differences in the shape and basal cAMP-PKA signaling, changes in local cAMP levels recorded with FRET sensors in cultured SANCs might be different from those in freshly isolated SANC. Moreover, because cAMP levels in the vicinity of individual PDEs could be relatively low (∼100 nmol/L), it might be beyond detection by currently available FRET sensors (Koschinski and Zaccolo, 2017). Recently developed AKAP79–CUTie FRET, however, shows some promise in this regard, and detects statistically significant changes of cAMP in the range of 100 nmol/L^-1^ μmol/L (Koschinski and Zaccolo, 2017). In short, future studies that utilize advanced methods of local cAMP measurements are required to understand specific mechanisms of compartmentalized PDE-regulated signaling and synergism of dual PDE3 + PDE4 inhibition in cardiac pacemaker cells.

## Author Contributions

TV made substantial contributions to conception, design and writing of the manuscript, and approved the last version for publication. EK helped to draft the manuscript, revised its content, and approved the last version for publication. EL helped to draft the manuscript, critically reviewed its content, and approved the last version for publication.

## Conflict of Interest Statement

The authors declare that the research was conducted in the absence of any commercial or financial relationships that could be construed as a potential conflict of interest.
